# Bridging the Gap between Theory and Ecology in Evolutionary Models

**DOI:** 10.1371/journal.pbio.0040405

**Published:** 2006-11-07

**Authors:** Liza Gross

The history of life is filled with examples of one species diverging into several, even thousands, each with unique traits geared to the demands of its ecological niche. In the textbook case of adaptive radiation, an ancestral finch species landed on the Galapagos Islands just a few million years ago, and evolved into 13 new species with specialized beaks adapted to exploiting the various seeds, nuts, insects, and other food sources on the island.

Adaptive radiations suggest that species evolution follows the first rule of business: find a niche and fill it. But that’s not what most models used to detect evolutionary patterns of trait evolution assume. And in a new study, Robert Freckleton and Paul Harvey demonstrate the limitations of that choice. They also introduce a method to minimize those limitations by using a diagnostic tool that can detect evolutionary patterns that deviate from the standard models.

The complexity of evolutionary processes and spottiness of the fossil record calls for statistical models—whose accuracy depends on their assumptions—to infer historical patterns of evolution. Traditional approaches to studying the evolution of traits (such as beak shape) typically compare populations, species, or higher taxa to identify adaptations and the corresponding evolutionary processes. With advances in molecular genomic techniques, comparative methods increasingly incorporate phylogenetic analyses, which compare gene or protein sequences to infer evolutionary relationships between taxa or traits.

These phylogenetic comparative methods often use a “Brownian motion” model of evolution, which assumes that more closely related species are more similar to each other and generate expected distributions of trait change among the species compared. Freckleton and Harvey suspected that the models could produce specious correlations, because they don’t explicitly account for ecological processes. Such a model—which, the authors point out, has rarely been tested—assumes (among other things) that traits evolve at a constant rate over time.

Freckleton and Harvey analyzed real and simulated data using a niche-filling model and a Brownian motion model and then applied two statistical tests as diagnostic tools to detect patterns of trait evolution that fall outside the assumptions of the Brownian motion. In the niche-filling model, niche space is initially empty (much like Darwin’s finches may have encountered), and new niches arise at a given rate, in random positions, and are instantly invaded by species with traits suited to exploiting that niche. Evolution occurs only when a new niche—such as a novel seed—appears and a species is under selection to exploit it. In contrast to Brownian models, for example, one would expect that as niches became filled with more species, the difference between the parent and offspring species would become smaller, because niches have a unique optimum value and trait values are constrained (by correlations between beak size and food size, for example). Likewise, with an adaptive radiation, one would expect ecological differences to arise with or shortly after speciation, rather than at a constant pace dependent only on time.

**Figure:**
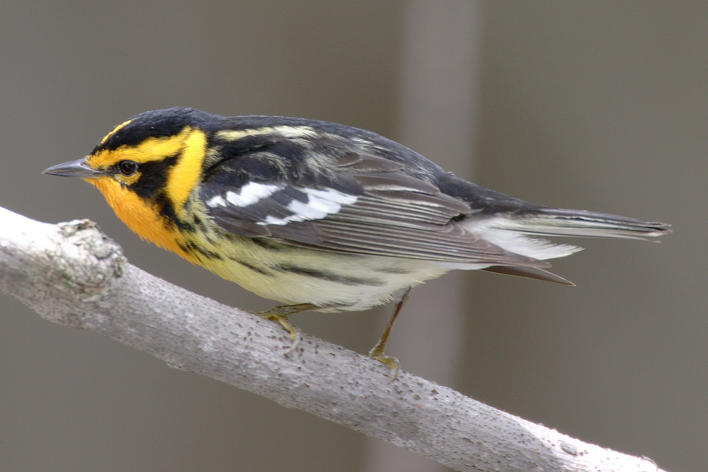
Old World Leaf warblers and Dendroica warblers (Dendroica fusca, pictured above), two classic cases of adaptive evolution, served as case studies for two diagnostic tests designed to reveal deviations from a Brownian motion model of trait evolution.

Speciation rate—defined as the rate at which new niches appear and are invaded in the niche-filling models and the rate at which lineages split in Brownian motion models—was modeled using three different models: the probability of speciation is proportional to the number of species present, remains constant, or declines with the number present. Each scenario reflects a different process corresponding to the invasion of an empty niche.

The two diagnostic tests included a “node height” test, which assesses whether the rate of evolution of a particular trait occurs systematically within a phylogenetic tree, and a simple randomization test. (Node height refers to the distance between the ancestral species, or root, and the most recent common ancestor for a pair under study.) In the latter case, if the data are consistent with Brownian motion, one would expect small and large changes of a particular trait (such as beak size) to be equally likely at any point in the phylogenetic history of the group of species compared .

The authors first used simulated data to provide statistical confidence levels for their two tests and showed that the power of each test to detect non-Brownian evolution depended on the model of speciation as well as the extent of correlation between traits. They then applied the tests to published data on the phylogeny and feeding habits of two warblers, both classic cases of adaptive radiation. Both statistical tests were able to detect non-Brownian evolution of two feeding-related traits (body size and prey size) in Old World Leaf warblers. In a second case, neither test detected deviations from the Brownian model for the evolution of beak shape and size in Dendroica warblers—indicating that Brownian motion correctly described the pattern of trait evolution in this case, which provided a case study for the alternative scenario.

The authors emphasize the diagnostic nature of these tests and the need for developing more-refined techniques to detect deviations from Brownian evolution. But their results underscore the importance of incorporating ecological processes into comparative models, to provide a more realistic and detailed account of the historical pressures and mechanisms driving the diversification of life.

